# Mechanical properties and acoustic emission analysis of desert sand concrete reinforced with steel fiber

**DOI:** 10.1038/s41598-022-24198-2

**Published:** 2022-11-28

**Authors:** Changwei Qu, Yongjun Qin, Ling Luo, Liangliang Zhang

**Affiliations:** College of Civil Engineering and Architecture, Xinjiang University, Urumqi, 830017 China

**Keywords:** Civil engineering, Mechanical engineering

## Abstract

In this study, to reduce the consumption of natural sand and improve the utilization rate of desert sand in western China, while preparing 14 groups of samples, desert sand is used to replace natural sand by the ratio of 20%, 40%, and 60%, and steel fiber is mixed with volume fraction 0.5%, 1.0%, 1.5%, and 2.0%. The mechanical properties of the specimens, including compressive strength, splitting tensile strength, and axial compressive strength were tested. Besides, the microstructures of the samples were analyzed by SEM, XRD, and acoustic emission detection technologies to identify the damage process. The results show that the desert sand can refine the microstructure and fill the pores, and it has good comprehensive properties at a 40% substitution rate. The compression properties of specimens are not apparently improved, but the tensile strength and deformation properties are significantly improved. The steel fiber with 1.5 vol% content behaves better, and the 28d compressive strength of the optimized group reaches 58.7 MPa. As a result, the polynomial fitting degree of total AE hits and stress level receives a more incredible goodness (R^2^) value than 0.96. The strength characteristics of steel fiber-desert sand concrete (SFDSC) can meet the demands of C40 concrete, and this research can provide a reference for engineers using desert sand in their designs.

## Introduction

In recent years, the problem of carbon emission has been gradually focused on, and low-carbon green building materials have become the focus of research in the industry^1^. With the increasing demand for building materials, river sand begins to show the phenomenon of running out or even depleting^2^, and the massive mining of river sand has also brought severe environmental problems such as undercutting riverbeds and loss of sand and gravel^3^. Furthermore, there is a large number of desert areas around the world, which are formed by weathering of parent rocks; the sand in these areas has a low fineness modulus and high water demand, making it challenging to apply desert sand to cement^4^. In addition to alleviating the scarcity of river sand, the efficient utilization of desert sand will provide a reference basis for desert management in local areas.

The critical reason for limiting the application of desert sand resources is the low fineness modulus, high water requirement, and concrete bleeding problem. Most studies have concluded that desert sands are finer-grained and play a good role in filling and improving the density of the paste^5–8^, which can improve the mechanical properties of concrete in a reasonable mix ratio^9^. Mixing mineral admixtures^10,11^ and fiber reinforcement^12–14^ with desert sand concrete (DSC) has also been found to improve performance, which provides a practical reference to overcoming the DSC’s shortcomings. On the other hand, the properties of desert sand are different from river sand; it contains small amounts of alkali metal oxides such as K_2_O and Na_2_O, and active elements such as Si, Al, and Ca, which will produce potential hydration products to enhance the concrete properties^15^. However, the chemical composition and fineness modulus of desert sands are different in different regions^16^, affecting the universal law of desert sand materials^17^. Meanwhile, acoustic emission techniques have started to use in the damage detection of concrete, but concrete damage monitoring of compounds with steel fibers and desert sands has been relatively underreported.

According to previous research, it can be known that concrete has poor tensile properties, and in order to increase its ductility, fibers are added to concrete for reinforcement^18^. According to available research^19–23^, adding fibers can inhibit the development of microcracks and restrain the tensile strain after cracking; which is an effective way to improve the mechanical (strength and shrinkage) properties of concrete, but researchers^24,25^ also claim there was a decrease in compressive strength after incorporation of fibers. Meanwhile, fiber distribution influences the strength of concrete^26^, which is in turn impacted by the matrix rheological properties^25,27^. Thus, the matrix’s quality and the fibers’ properties (type, volume, aspect ratio, etc.) will impact concrete performance^28^. As fiber reinforcement has been effective at improving the load-bearing capacity of concrete, it could be a feasible way to enhance the performance of desert sand concrete by adding fiber as well as improving the utilization of desert sand in the local area.

Therefore, considering the convenience of obtaining materials in Xinjiang and the urgent need for environmental management in sandy areas, Taklamakan desert sand is selected in this paper to prepare samples and test the mechanical properties of 14 groups with different desert sand replacement rates (*R*_ds_) and steel fiber contents (*V*_sf_). The concrete axial compression process is characterized by the acoustic emission (AE) technique to analyze the fitting of total AE hits to stress levels. As a final step, SEM and XRD were performed to provide analytical verification of the microstructure. This study aims to provide an experimental basis for using desert sand resources as construction materials, which will also serve as a valuable guide for better understanding the characteristics of fiber and desert sand synergy, improving the effective utilization of desert sand resources, and facilitating local use in desert areas.

## Materials and methods

### Materials

**Figure 1:**
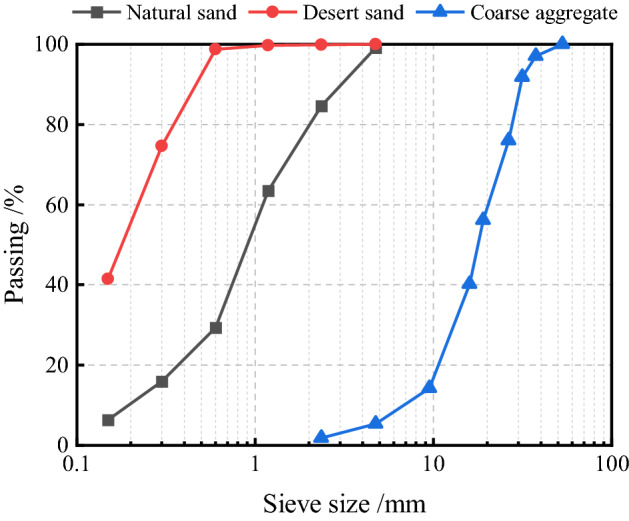
Particle distribution of coarse and fine aggregate.

P.O 42.5R portland cement was selected for all concrete mixtures. The desert sand was collected from the Taklamakan desert, with an average particle size of 0.963 mm, and fineness modulus of 0.855; its chemical composition is shown in Table 1. The medium sand in Xinjiang, with a fineness modulus of 2.99 and an apparent density of 2487.5 kg/m^3^, was chosen as a natural fine aggregate. The coarse aggregate was made of natural pebbles with a continuous gradation of 5–20 mm, and its bulk density is 2700 kg/m^3^. Figure 1 shows the particle distribution of the aggregates. The hooked steel fibers are produced in Hebei, China, and the specific parameters are shown in Table 2. The retarder is a Q8081 polycarboxylate superplasticizer, and the water reduction rate is ≥ 25%.

**Table 1:** Chemical composition of materials (mass fraction)/%.

Type	CaO	SiO_2_	Al_2_O_3_	Fe_2_O_3_	SO_3_	MgO	R_2_O	K_2_O	Na_2_O	Loss
Cement	56.78	25.52	7.51	2.89	2.43	1.33	0.93	0.67	0.49	1.45
Natural sand	0.11	90.76	4.59	0.73	0.05	0.18	–	2.16	0.39	0.87
Desert sand	9.56	55.61	4.77	2.44	0.05	2.32	–	2.54	2.08	10.38

**Table 2:** Basic parameters of hooked steel fiber.

Type	*L*_0_/mm	*L*_f_*/*mm	*D*_f_*/*um	*L*_0_/*D*_f_	*f*_tk_/Mpa
Hooked steel fibers	35	38	750	46.7	1000

*L*_0_ is effective length; *L*_f_ is physical length; *D*_f_ is the effective diameter; *f*_tk_ means tensile strength.

### Preparation of concrete

Based on the previous research^29–31^, this paper focused on the effect of desert sand replacement rate (*R*_ds_) and steel fiber content (*V*_sf_) on the performance of desert sand concrete (DSC), fiber content by volume and concrete design strength of C40. With a detailed mix design shown in Table 3, the superplasticizer dosage was adjusted based on fluidity during trial formulation to meet slump requirements. The samples were prepared according to the procedures specified in Chinese standards (GB/T 50081-2019)^32^ and (CECS 13-2009)^33^.

**Table 3:** Mix design of steel fiber reinforced desert sand concrete/(kg m^−3^).

Group	*R*_ds_/%	*V*_sf_/%	Cement	Fiber	Superplasticizer	Water	Desert sand	Fine aggregate	Course aggregate
JZ-0	0	0	325	0	1.46	146	0	768	1152
JZ-1	20	0.5		39	1.46		153	614	
JZ-2	20	1.0		78	1.46		153	614	
JZ-3	20	1.5		117	1.46		153	614	
JZ-4	20	2.0		156	1.46		153	614	
JZ-5	40	0.5		39	1.46		307	461	
JZ-6	40	1.0		78	1.46		307	461	
JZ-7	40	1.5		117	1.46		307	461	
JZ-8	40	2.0		156	1.46		307	461	
JZ-9	60	0.5		39	1.31		461	307	
JZ-10	60	1.0		78	1.31		461	307	
JZ-11	60	1.5		117	1.31		461	307	
JZ-12	60	2.0		156	1.31		461	307	
JZ-13	40	0		0	1.46		307	461	

*R*_ds_ means replacement rate of desert sand; *V*_sf_ means volume content of steel fiber.

**Figure 2:**
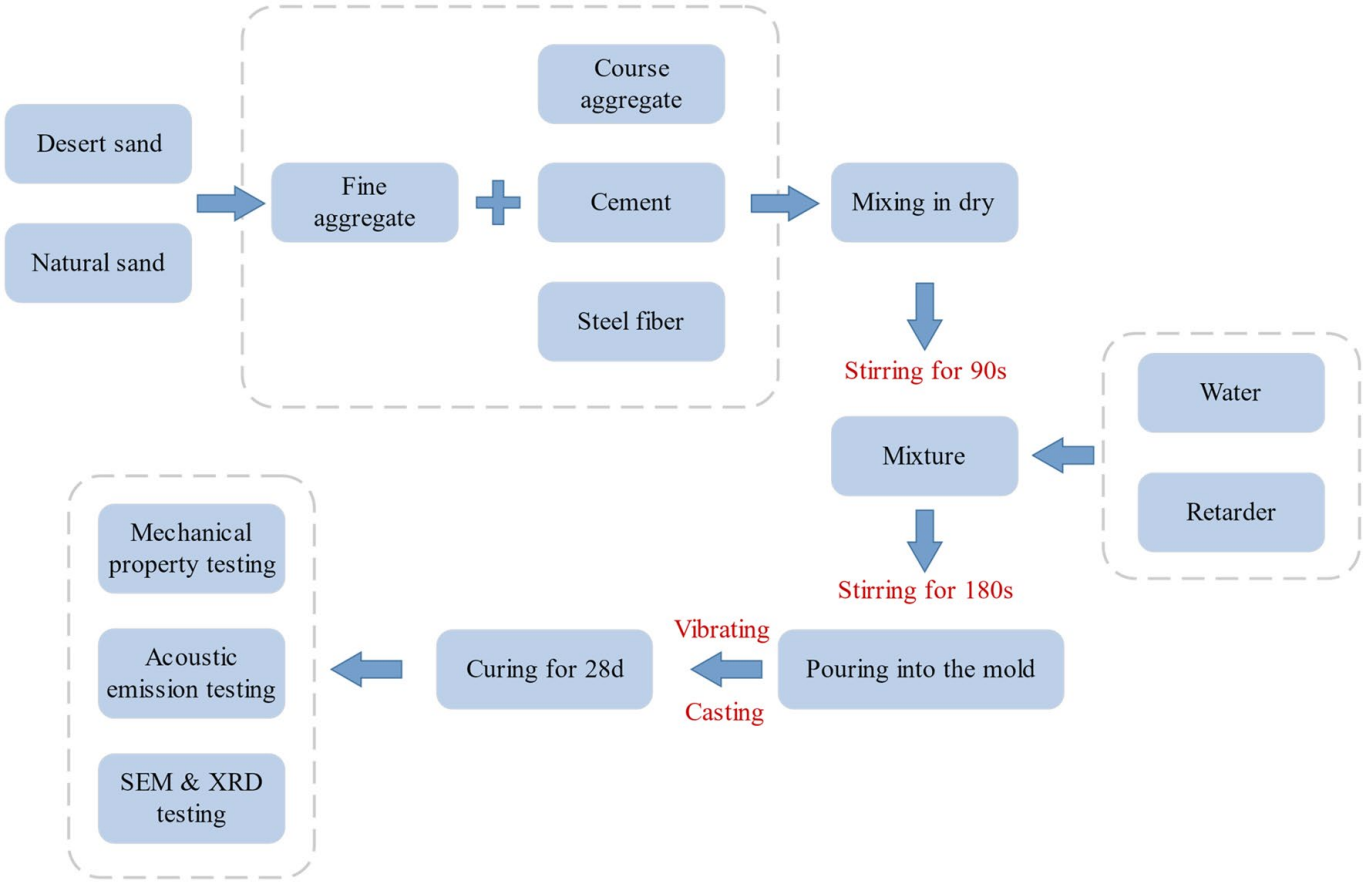
Test flowchart of steel fiber desert sand concrete.

According to Table 3, the water-to-binder ratio (w/b) was fixed at 0.45, the sand-to-aggregate rate was 40%, and the superplasticizer dosage was about 0.5% of the cementing materials. In the mixture, desert sand partially replaces natural sand, and steel fiber is added by volume content. As is shown in Fig. 2, after weighing coarse aggregate, fiber, fine aggregate, and cement, they were mixed for 90 s to ensure uniform dispersion, then retarder and water were added and mixed for another 180 s; finally, the mixtures were poured under vibration into 150 mm * 150 mm * 150 mm cube and 150 mm * 150 mm * 300 mm prismatic molds, and were demolded after keeping 24 h at 90% RH/20 ℃ and final curing for 28 days.

### Methods

**Figure 3:**
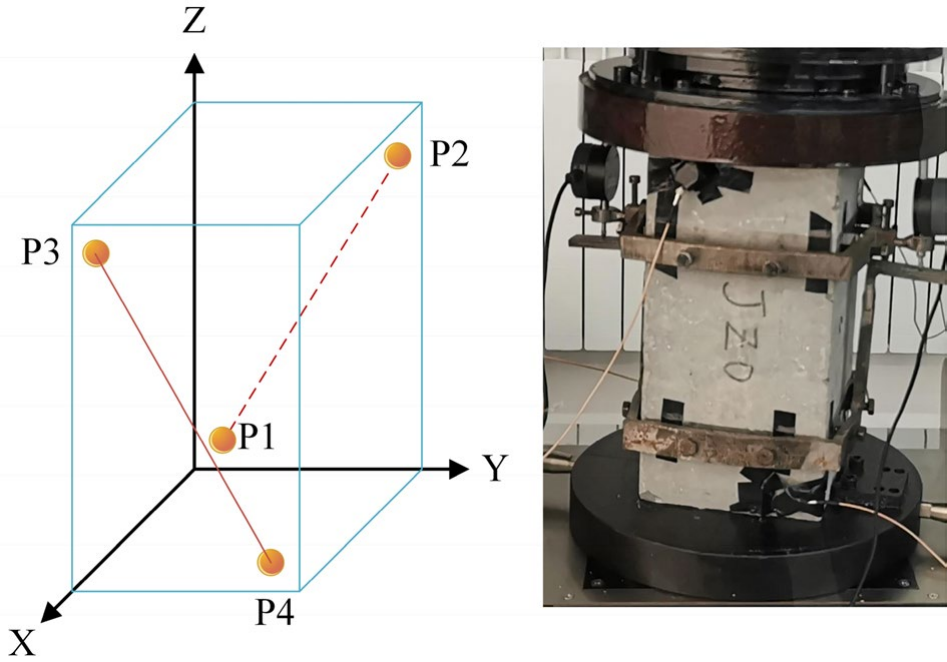
Schematic diagram of acoustic emission test.

After being cured to the age of 28d, samples were removed from the curing room and cleaned of surface debris and water before being tested for mechanical properties. The test parameters include compressive strength, splitting tensile strength, axial compressive strength and stress–strain curves. All steps were performed conforming to the Chinese standards norms (GB/T 50081-2019). The compressive strength of the DSC was tested by the compressive machine (WHY-3000) with a loading speed was 0.06 MPa/s. The axial compressive strength was tested by the compressive machine (306A-3000) with the method of displacement controlling, and the loading speed was 0.1 mm/min; the axial compression process using the acoustic emission acquisition instrument (SAEU2S) for the measurement and acquisition of its internal damage data. In all of test samples, the maximum and minimum values shall not exceed 15% of the average. Figure 3 shows the test schematic and AE probe arrangement. A steel collar is used to fix the prismatic sample, and the axial displacement is measured by a micrometer. Then AE probe and test point contact parts are coated with petroleum jelly and taped after wiping the surface of the samples.

## Results and discussions

### Mechanical properties

**Figure 4:**
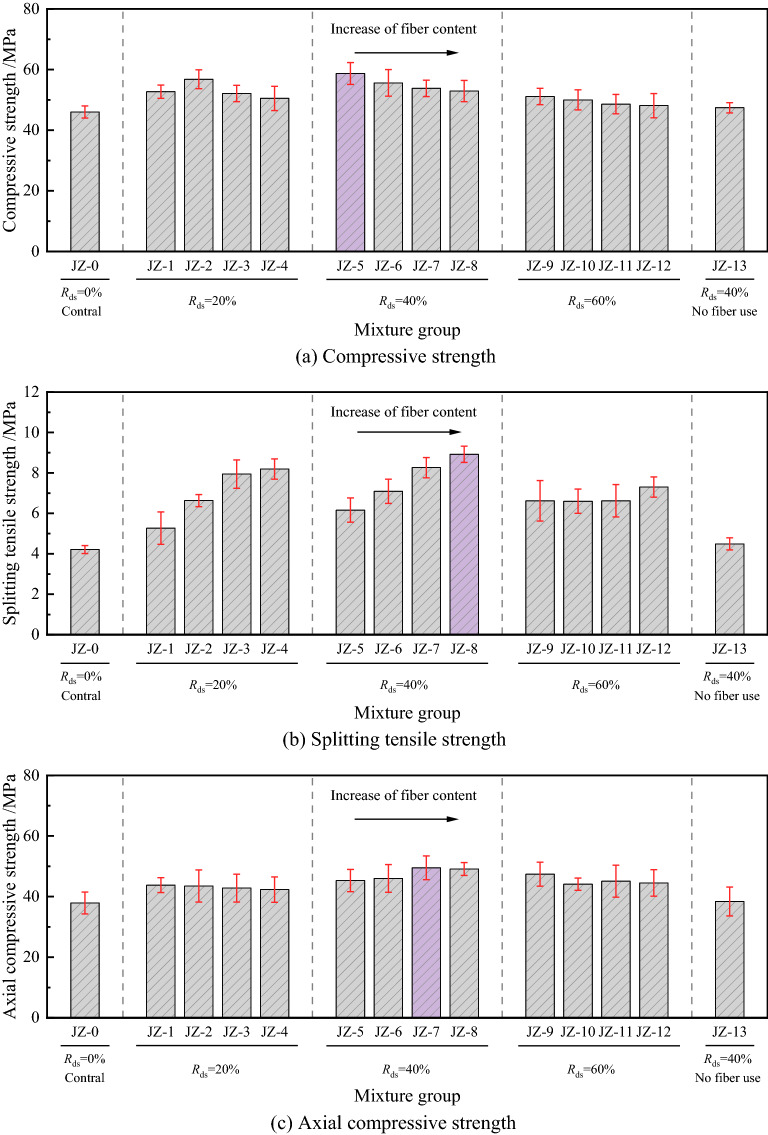
Mechanical properties of concrete with different *R*_ds_ and *V*_sf_.

Figure 4 shows the mechanical properties of each group at different *R*_ds_ and *V*_sf_. Overall, the performance of *R*_ds_ at 40% is better, and there are slight differences in the performance between samples at 20% and 60% replacement rates, but all their strength has exceeded JZ-0. The addition of steel fibers showed a more significant improvement in splitting tensile strength; this may benefit from the fibers’ bridging effect, which effectively limits the development of inside microcracks during the damage process. The fiber content, however, was not found to improve compressive strength significantly, and Xu et al. also drew similar conclusions in their study^34^. In the following sections, we will discuss the mechanical properties separately.

#### Compressive strength

**Figure 5:**
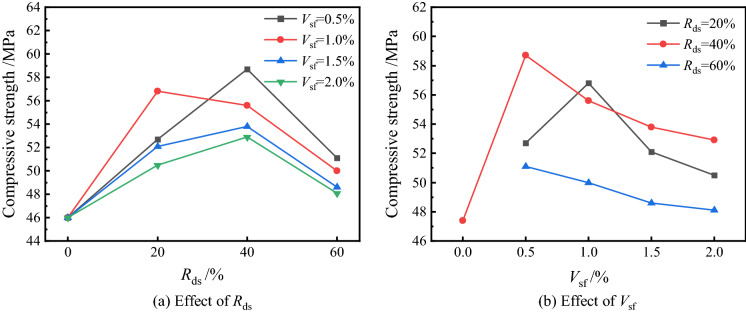
Effect of different *R*_ds_ and *V*_sf_ on compressive strength.

Figure 5 demonstrates the effect of different *R*_ds_ and *V*_sf_ on the compressive strength of DSC. It can be found that the highest strength of all groups was JZ-5 at *V*_sf_ = 0.5%/*R*_ds_ = 40%, and was an increase of 27.6% compared to the JZ-0 compressive strength that was increased from 46 to 58.7 MPa. At the same *V*_sf_, the compressive strength showed a trend of increasing and then decreasing with increased of *R*_ds_. In the group at *V*_sf_ = 1.0%, only the JZ-2 reached the highest strength of 56.8 MPa, which was increased by 23.5% compared to JZ-0, and other groups reached their peak at *R*_ds_ = 40%. The results showed that *R*_ds_ = 40% could present better strength properties, which is consistent with the findings of Lv et al.^35^. As an extra fine aggregate with a low fineness modulus, desert sand can fill the internal pores of the concrete and improve its compactness. Although desert sand has a large surface area and high water demand, it might absorb part of the water and play a role in dispersing colloids, which may positively impact mechanical properties^11^. On the other hand, even though the desert sand from the weathered parent rock has a more stable crystalline nature, its composition contains a certain amount of CaO and reactive Al_2_O_3_, which still shows some volcanic ash reactivity in the hydration reaction, promoting the generation of the hydration product C–S–H gel and improving the compressive properties of the matrix^36^. However, the high replacement perhaps reduces concrete workability. To achieve the slump requirement, we may need to increase water content, which may cause water secretion or not be easy to vibrate densely, thereby reducing performance.

It can be seen from Fig. 5b that considering the effect of *V*_sf_, the compressive strengths of JZ-5 to JZ-8 are 58.7 MPa, 55.6 MPa, 53.8 MPa and 52.9 MPa, respectively; for the same *R*_ds_, the compressive strength gradually decreases with *V*_sf_, and the strength was increased by 23.8%, 17.3%, 13.5% and 11.6% respectively compared with 47.4 MPa of JZ-13. However, an anomalous set of data was also observed, where the JZ-2 group showed an increase in strength when the *V*_sf_ was increased to 1.0%, then decreased in line with the trend, and the rest of the groups showed a decrease in strength with the increase in fiber content. It can be concluded that at lower *V*_sf_, it is favourable to the compressive strength, but after exceeding 1.0%, the interfacial transition zone (ITZ) with weak mechanical properties will be formed on the surface of the fiber-to-paste and fiber-to-aggregate. Under stress, these parts will crack rapidly and gradually connect to form damaged cracks, resulting in a certain degree of deterioration of compressive strength^37^.

#### Splitting tensile strength

**Figure 6:**
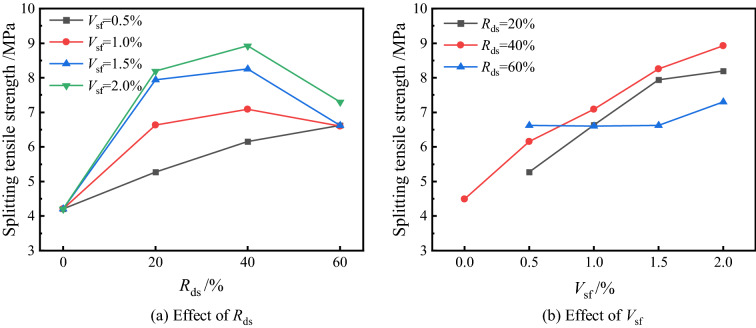
Effect of different *R*_ds_ and *V*_sf_ on splitting tensile strength.

As shown in Fig. 6, the splitting strength of the specimens roughly showed an increasing trend in the first and then a decreasing trend for all fiber content, and only for *V*_sf_ = 0.5%, the splitting strength was enhanced with the increase of *R*_ds_. For all groups of fiber content, when *R*_ds_ = 20%, the strength was increased by 25.2%, 57.5%, 88.6% and 94.5% compared to JZ-0, respectively, and the overall strength improvement trend increased with *V*_sf_. In terms of the highest strength set of curves (*V*_sf_ = 2.0%), as the *R*_ds_ increased, the splitting strength of the specimens reached 8.19 MPa, 8.92 MPa, and 7.30 MPa successively, and all groups increased at other *V*_sf_ levels. The matrix still contributes to the tensile strength of concrete even though its tensile properties are weak. During the test, uniformly distributed tensile stresses are generated in the compressed centerline, and the bearing capacity increases as the strength of the matrix increases. Nevertheless, special data points were observed in which the *V*_sf_ increased from 0.5 to 1.5% when *R*_ds_ = 60% and the error limits of the data were larger; the strengths of the three groups were relatively close to each other without an obvious pattern. This phenomenon is attributed to the ultrafine properties of desert sand. Based on previous studies^38^, it is considered that desert sand within a certain replacement rate can ensure stable development of the strength of concrete; when *R*_ds_ is too high (> 50%), the mixture cannot form a uniform particle gradation, which may lead to instability in the strength of the samples.

Unlike the aforementioned compressive strength law, the enhancing effect of fibers on the splitting tensile strength is very significant, which can effectively act as “micro reinforcement” to disperse the stresses during the tensile cracking process as the steel fibers are distributed in a 3D disorderly manner within the matrix, thus increasing its contribution to the bearing capacity^19^. For the tensile strength enhancement, after matrix cracking, the fibers can effectively bear the tensile stress and better inhibit the development of microcracks^39,40^. Some studies have pointed out that the distribution of fibers may be affected by the yield stress, and the concrete performance may decrease due to an inadequate orientation of fibers^25^. By contrast, the hooked fibers improve the contact resistance between the two parts, and this form of embedding is similar to the anchorage of a reinforcement bar and greatly enhances the integrity of the fibers and the concrete.

#### Axial compressive strength

**Figure 7:**
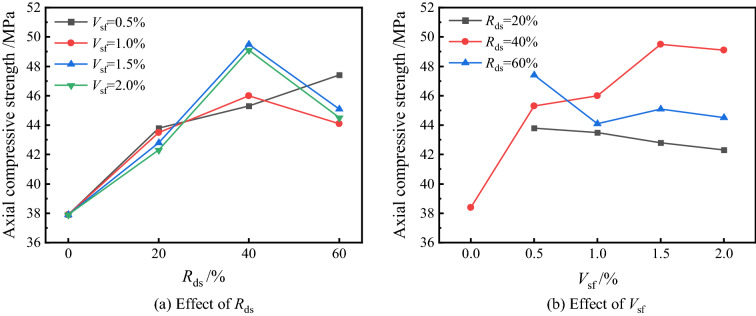
Effect of different *R*_ds_ and *V*_sf_ on axial compressive strength.

The trend of axial compressive strength is similar to that of cubes, but the effect of factors is different due to the change in sample size. Figure 7 shows the results of axial compressive strength, and it is easy to see that with the increase of *R*_ds_, the axial compressive strength also shows the trend of increasing first and then decreasing and better mechanical properties when *R*_ds_ = 40%. The strength of each group reached 42.3–43.8 MPa without much fluctuation when *R*_ds_ = 20%. When the replacement rate was increased to 40%, the trend of each group showed unconformity, and the strength of JZ-5 at *V*_sf_ = 0.5% was only 45.3 MPa, which was 3.4% higher than 43.8 MPa of JZ-1, while with an increase of 15.7% and 16.1% the strength of JZ-7 and JZ-8 were more obviously increased to 49.5 MPa and 49.1 MPa, respectively. When the *R*_ds_ was increased to 60%, only the strength of the JZ-9 still increased to 47.4 MPa, which was 4.6% higher than that of JZ-5, while a decrease in strength was observed in other groups. The results show that the fibers and the matrix resist stress together during the concrete compression process, but the lower tensile bearing capacity of the concrete during the tension process leads to tiny cracks first; at the same time, the stress is transferred to the fibers, thus limiting the further development of the cracks. This can lead to lower matrix performance and affect the axial compressive strength at higher *R*_ds_. Due to the fine particle size and high specific surface area of desert sand, a higher admixture will result in weaker parts of the matrix, decreasing mechanical properties^41^, which is consistent with the compression strength law. On the other hand, among the different loading methods in the uniaxial compression test, the displacement control, makes the internal damage have enough time to develop. In addition, the "confinement effect" during the loading process constrains the sample along the direction of tensile stress.

The fiber has a negative effect on the axial compressive strength at the high volume dose, but unlike the cube compressive strength law, it shows an increase in the axial compressive strength at *V*_sf_ = 1.5%. However, this pattern may be related to the randomness of fiber distribution. Perpendicular distribution of fibers to the compression direction can effectively disperse the tensile stress and limit the further development of microcracks by their bridging action^42^. It can be seen from Fig. 6b that the highest axial compressive strength was obtained when *R*_ds_ = 40%/*V*_sf_ = 1.5%, followed by *R*_ds_ = 60% and *R*_ds_ = 20%. Among the three curves, the 40% replacement rate shows a gradual increase in strength with increasing *V*_sf_, and the strength starts to invert after the *V*_sf_ exceeds 1.5%, which is related to the ITZ inside the matrix. The remaining two curves largely show a negative correlation trend, where the strength at *R*_ds_ = 20% is lower, its strength development is influenced by the concrete, and the compressive strength bearing capacity contribution is mainly based on the cement matrix. At the same time, the fibers mainly play a crack-arresting role and have little contribution in enhancing the compressive bearing capacity^43^.

#### Stress–strain relationship

**Figure 8:**
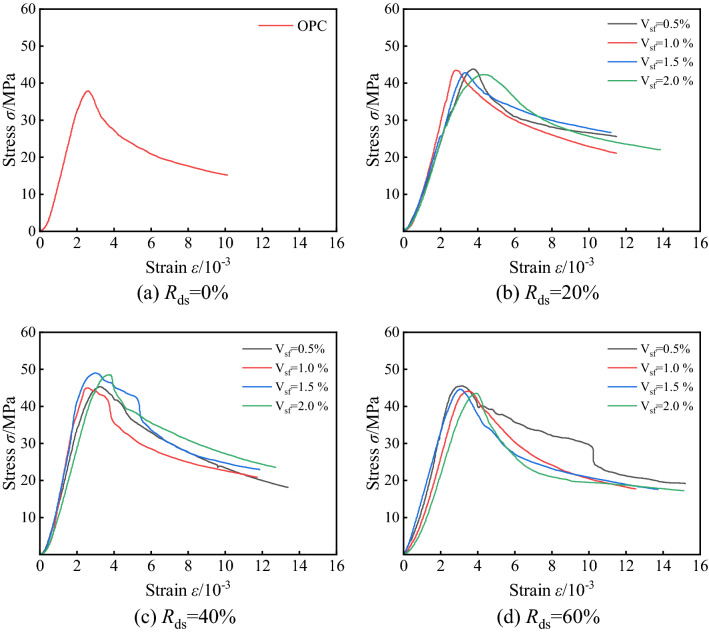
Stress–strain curves of SFDSC under uniaxial compression.

Figure 8 shows the axial compressive stress–strain curve of steel fiber-desert sand concrete. It can be seen that the *V*_sf_ does not show an obvious effect on the peak stress at the same *R*_ds_, but it improves the concrete ductility and the falling section after the peak stress tends to be moderate. In Fig. 8d, it is observed that the *V*_sf_ = 0.5% has an abnormal curve in the descending section. After the curve reaches the peak stress, it drops briefly and then appears as a slight rebound; there is a significant fluctuation in the stress–strain curve; the strain drops steeply when ε reaches 10 × 10^–3^, and local damage occurs in a very short time with a large amount of skin peeling off the surface and rapid crack occur. This situation indicates the unstable performance of DSC under a high replacement rate, the desert sand cannot play a suitable filling effect after the change of particle gradation, and the matrix compactness has defects. The highest peak stress was found in all samples when *R*_ds_ = 40%/*V*_sf_ = 1.5%, reaching 49.5 MPa; compared with other samples, the average value of peak strain of fiber-reinforcement DSC was 0.0033, which was greater than the peak strain of 0.0026 for Ordinary Portland cement concrete (OPC); the average value of ultimate strain was 0.0052, which was greater than the peak strain 0.0032 (Table 4). The specimens also demonstrated a greater destabilization strain than OPC, and the fibers bridged the stresses and delayed crack damage during crack development, resulting in better ductility. According to the above conclusion, after incorporating steel fibers, DSC has a better deformation capacity than OPC.

**Table 4:** Characteristic value statistics of the stress–strain curve.

Group	*R*_ds_/%	*V*_sf_/%	***σ***_max_/MPa	***ε***_**0**_	***ε***_**cu**_	***ε***_**F**_
JZ-0	0	0	37.9	0.0026	0.0032	0.0100
JZ-1	20	0.5	43.8	0.0038	0.0030	0.0173
JZ-2	20	1.0	43.5	0.0024	0.0049	0.0108
JZ-3	20	1.5	42.8	0.0033	0.0047	0.0112
JZ-4	20	2.0	42.3	0.0040	0.0065	0.0134
JZ-5	40	0.5	45.3	0.0032	0.0047	0.0133
JZ-6	40	1.0	46.0	0.0021	0.0050	0.0113
JZ-7	40	1.5	49.5	0.0025	0.0059	0.0116
JZ-8	40	2.0	49.1	0.0034	0.0076	0.0163
JZ-9	60	0.5	47.4	0.0036	0.0053	0.0155
JZ-10	60	1.0	44.1	0.0035	0.0047	0.0124
JZ-11	60	1.5	45.1	0.0031	0.0040	0.0137
JZ-12	60	2.0	44.5	0.0047	0.0054	0.0198
JZ-13	40	0	38.4	0.0034	0.0058	0.0159

*R*_ds_ means replacement rate of desert sand; *V*_sf_ means volume content of steel fiber; ***σ***_max_ is peak stress; ***ε***_**0**_ is perk strain; ***ε***_**cu**_ is ultimate strain, take the value at 0.85***σ***_max_ in the descending section of the stress–strain curve; ***ε***_**F**_ is the strain when the specimen is unstable.

### Acoustic emission damage analysis

#### Damage appearance

**Figure 9:**
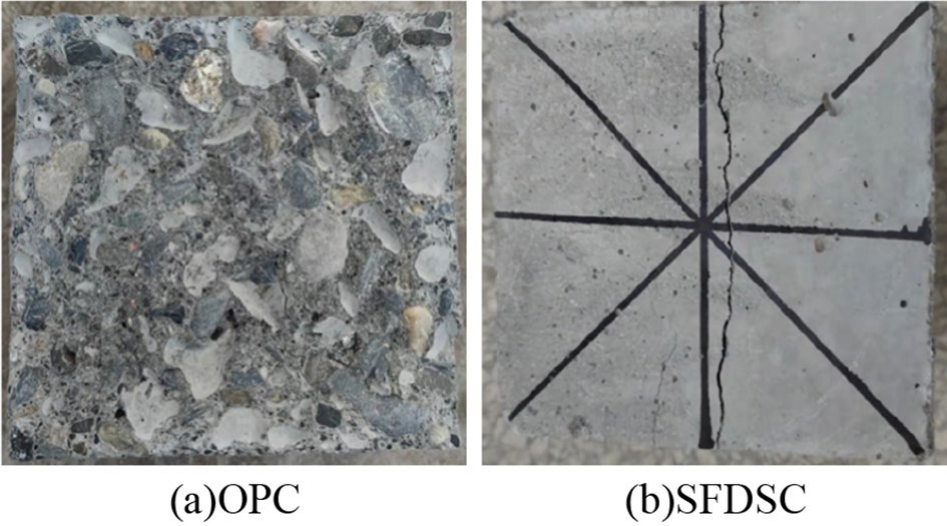
Photos of concrete splitting tensile strength test.

**Figure 10:**
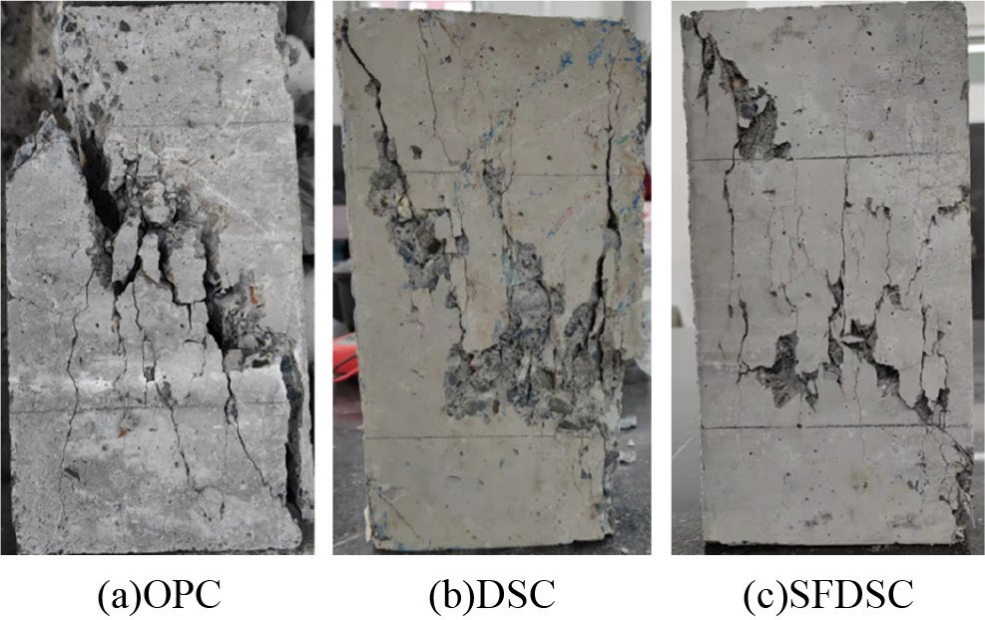
Photos of concrete axial compressive strength test.

Figure 9 shows the splitting tensile test damage; OPC will break into two pieces under ultimate load, while steel fiber can guarantee the concrete's integrity; cracks appear and gradually penetrate under load but will not brake suddenly. Figure 10 shows the axial compressive test damage; as it was shown, fiber improves strength because the tensile properties of steel fiber are much higher than concrete. In the process of the crack appearing, the concrete cracked in tension fast, and then the steel fiber began to bear the tensile stress, forming the crack area near the stress redistribution. So the load continues to increase, and the steel fiber limits the development of the main crack, in its surroundings occur small and dense microcracks after concrete begins to appear skin spalling. As mentioned above, steel fibers improve the tensile properties of concrete and increase their ductility, which is extremely important for structural safety.

#### Acoustic emission

**Figure 11:**
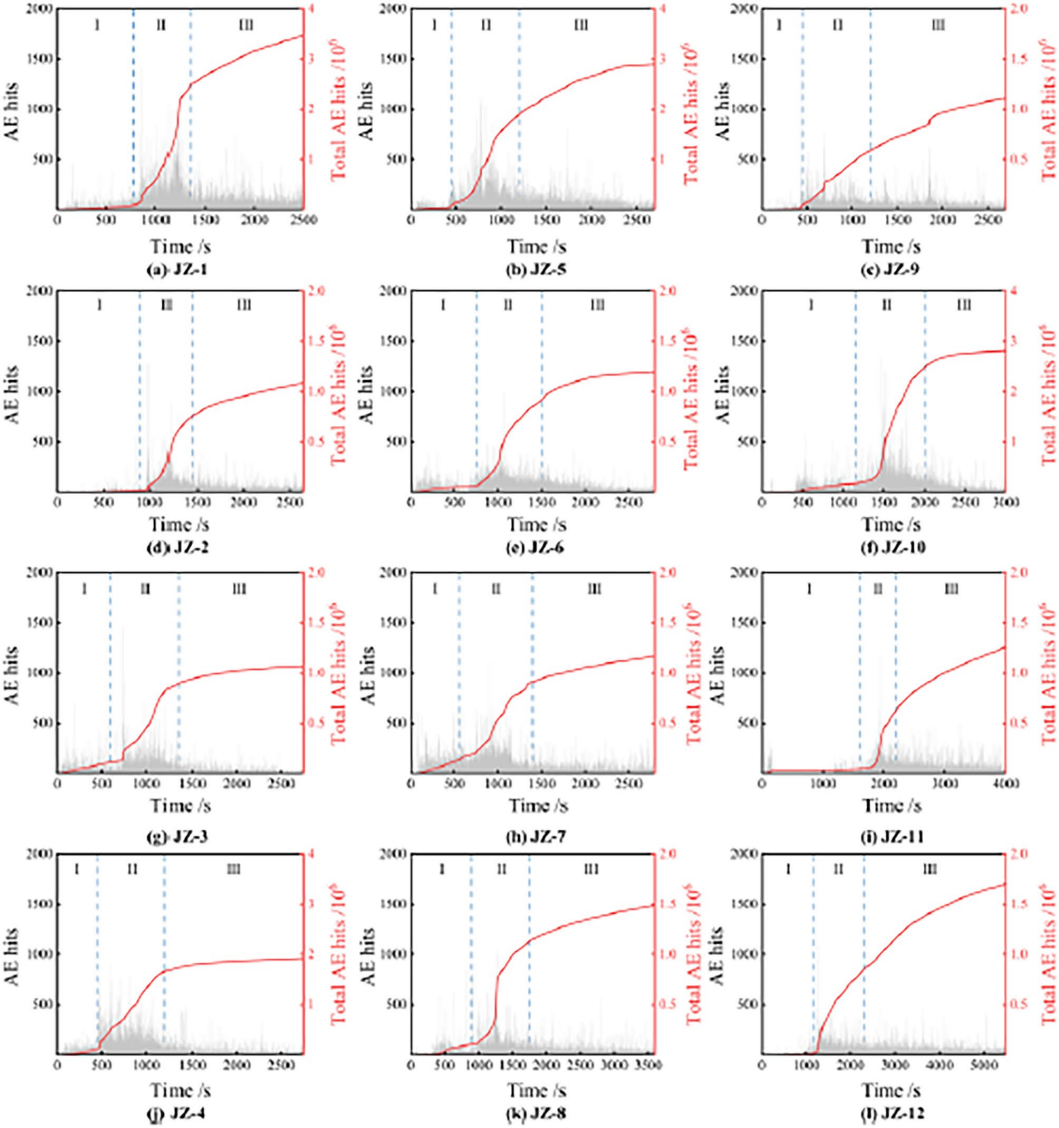
AE hits and time correlation statistics of each group.

In addition to the visual inspection, this paper adopts acoustic emission technology to monitor the process of concrete axial compression. When damage occurs, the acoustic emission event is statistics once exceeding the threshold number, which is one AE hit to reflect the change of damage inside the concrete. Figure 11 shows the time-AE hits statistics and is divided into three stages: initial stage (I), rapid rise stage (II) and stable stage (III). Among these three stages, the samples in the initial stage are still in the stage of online elastic change and elastoplastic change, and the internal stresses are not enormous under small displacements, triggering fewer AE hits^44^. With the increase of displacement, the sample began to appear extrusion inside to compress the tiny pores and increase the degree of matrix compactness, the internal microcracks gradually began to develop under the action of stress, and the AE hits gradually increased. In the rapid rise stage, the steel fibers began to occur shear deformation, the concrete appeared to happen local crushing or surface spalling, and the damage intensified; at the same time, the AE hits appeared to rise steeply, at which time the sample was in a state close to the ultimate bearing capacity. As the displacement continues to increase, the damage occurs after the sample exceeds the ultimate bearing capacity, the stress level decreases, but the displacement increases rapidly; before the matrix loses bearing capacity and integrity, the AE hits begin to decrease, and the curve tends to level off. As the stress reaches its peak, the total AE hits show rapid growth, and the overall curve almost resembles an "S" shape, while the AE hits roughly follow a normal distribution with time, which aligns with the curve trend for stress–strain relationships.

As shown in Fig. 11, when the *R*_ds_ increase from 0 to 40%, the pores are easily filled by the extra fine aggregate, and matrix compactness will be improved so that damage is slight. However, when *R*_ds_ was further increased to 60%, the aggregate gradation was weakened and the internal structure deteriorated, resulting in the growth of total AE hits. Therefore, due to adding fiber, stress is transferred to the fiber during crack development to prevent further damage. Because of the influence of fiber distribution, the rapid rise stage (II) occurs between 500 and 2000 s, and AE hits in this stage will be jointly affected by various factors. The AE event record of crossing the threshold value is random, so individual samples will appear as regular anomalies, but it does not affect the general law.

**Figure 12:**
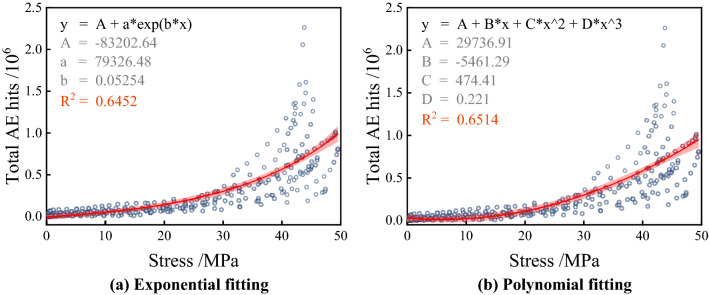
Fitting curve of total AE hits and stress.

**Figure 13:**
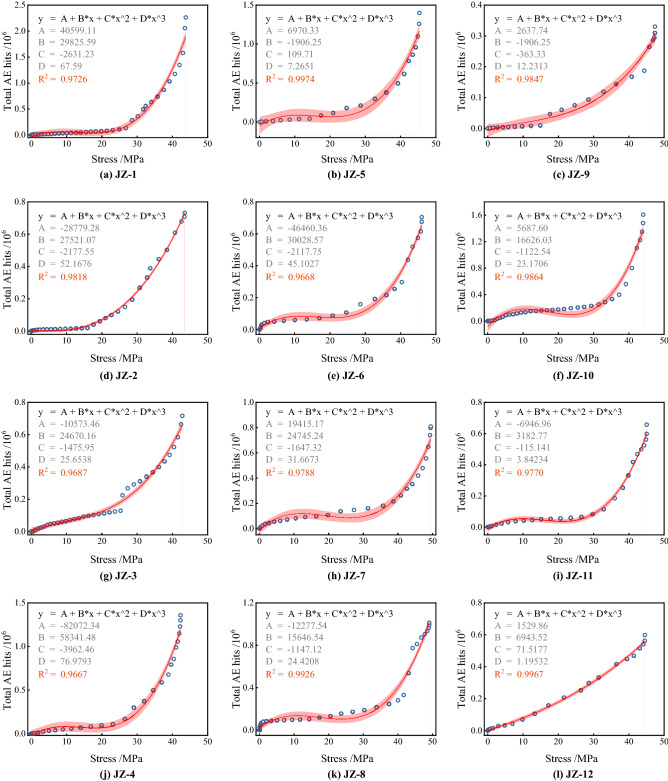
The fitting curve of total AE hits and stress in each group.

According to the relationship between AE hits and stress, two types of functions were selected for fitting. Figure 12 shows the fitting results of exponential and cubic polynomials, and it can be found that the cubic polynomial has higher fitting variance and higher accuracy. However, it is also found that as the stress increases, the total AE hits start to show dispersion; this is because that the total AE hits are affected by the mix design and fiber content, and only AE events above the threshold value can be recorded so that the response of the different group to the load varies at higher stresses^45^. In Fig. 13, the cubic polynomial fitting functions for each group are compared; specifically, it is found that the equations for a single group show a high fitting effect with R^2^ above 0.96. It can be concluded that the total AE hits show a positive correlation with the stress level, and as the stress continues to increase, the internal damage intensifies, and more AE events are triggered at this time.

### Microstructure

**Figure 14:**
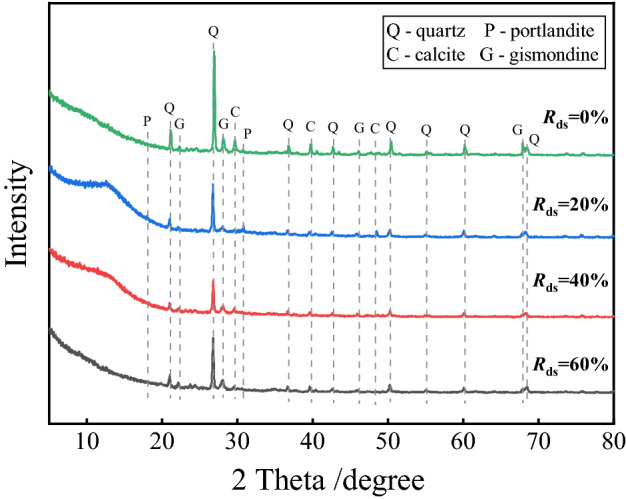
XRD patterns of different desert sand replacement rates.

Figure 14 shows the XRD of the samples at different *R*_ds_. In the DSC, the main silica phase is still the quartz, and it can be seen that the prominent peak of the quartz reaches the highest at 2θ = 26.77. With the increase of *R*_ds_, a more obvious change in the peak of quartz is observed, but this change does not have much effect on the hydration products. The silica content of the desert sand is lower than that of the normal sand, so the decrease in the quartz phase mainly results from increased *R*_ds_. In addition to the silica phase, the prominent diffraction peaks were plagioclase zeolite, calcite and calcium hydroxide stone, and a certain amount of aragonite was also observed, indicating that the samples had undergone a slight carbonation reaction while being cured to the test age. The DSC may have similar problems in terms of durability as OPC, which needs further study to explain.

**Figure 15:**
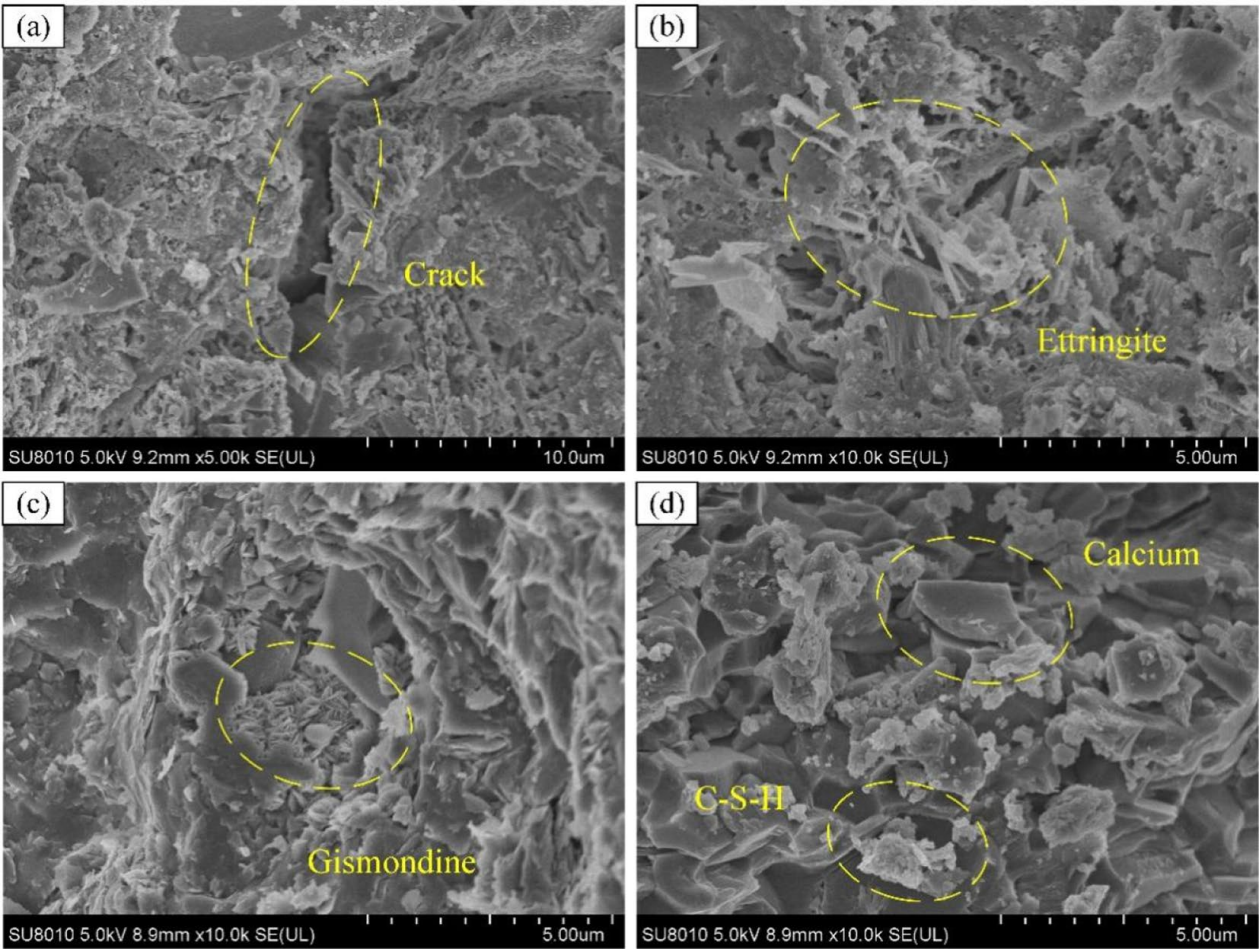
Scanning electron microscopy (SEM) image of desert sand concrete.

Figure 15 shows the SEM image of the DSC, and it can be seen that the physical phase structure is consistent with the XRD analysis; needle-like calcite, aragonite and other calcium carbonate phase products can be observed in the microcracks and broken surfaces. The cement hydration products are mainly flocculent C–S–H gel, the main source of matrix strength and somewhat determines the slurry properties. Hexagonal prismatic calcium hydroxide crystals, which make the unhydrated calcium hydroxide, will be a potential durability risk for carbonation and sulfate attack, whether DSC or OPC^46^.

## Conclusions

In this paper, the mechanical properties of desert sand concrete (DSC) with different desert sand replacement rates (*R*_ds_) and steel fiber contents (*V*_sf_) were investigated experimentally, and the process of concrete damage under pressure was monitored by the acoustic emission detection (AE) method. Combined with scanning electron microscopy (SEM) and X-ray diffraction analysis (XRD), the microscopic morphology of concrete was observed, which led to the following conclusions:

1. With the increase of *R*_ds_, the overall mechanical properties of the concrete show a trend of first increasing and then decreasing, and the reasonable *R*_ds_ can play a “micro-aggregate effect” to optimize the pore structure and improve the compactness of the matrix, realizing the best performance according to the test results *R*_ds_ = 40%.
2. Steel fibers have a significant effect on the splitting tensile strength of DSC, which is positively correlated with the *V*_sf_ due to the crack resistance effect of the fibers. However, the cement matrix contributes most of the concrete’s compressive strength, while fibers have a minor impact on the compressive strength.
3. After fiber incorporation, steel fiber desert sand concrete (SFDSC) exhibits a higher ductility than Ordinary Portland cement concrete (OPC), and the ultimate strain of concrete is increased, effectively increasing its bearing capacity. The falling section curve of fiber concrete slows down after the peak strain, and the strains at the damage time are all greater than those of OPC.
4. The SFDSC can meet the strength requirement of C40 concrete with the highest 28d compressive strength of 58.7 MPa. The optimum mix ratio is the JZ-7 group (*R*_ds_ = 40%/*V*_sf_ = 1.5%), and the compressive strength, splitting tensile strength, and axial compressive strength is 53.8 MPa, 8.26 MPa and 49.5 MPa, respectively.
5. Acoustic emission can provide a more intuitive description of the damage process. Total AE hits versus time curves reflect the damage process of concrete under stress, and AE thresholds are triggered as stress increases and damage occurs rapidly, with AE events tending to smooth out after peak stress conditions. There is a reliable positive correlation between total AE hits and stress level, the goodness of polynomial fitting can be more than 0.96.

## Data Availability

All data generated or analyzed during this study are included in this published article.
